# OP18 Shall We Pay More For Rare Disease Drugs? Assessing Decision-Makers’ Preferences Using A Sequential Discrete Choice Experiment Approach

**DOI:** 10.1017/S0266462325100767

**Published:** 2025-12-29

**Authors:** Yuanyuan Gu, Shan Jiang, Shunping Li, Haiyin Wang

## Abstract

**Introduction:**

Countries face challenges in prioritizing scarce health resources, often relying on evaluations based on quality-adjusted life years (QALYs). However, QALY gains may not fully capture intervention value, especially for rare disease treatments, which are often perceived as more valuable. This study examined whether Chinese health insurance decision-makers prioritize rare disease treatments and sought to quantify any additional value assigned.

**Methods:**

We conducted two sequential discrete choice experiments (DCEs). The first, labeled DCE, compared drugs for common and rare disease using five attributes—disease severity, childhood onset, catastrophic expenditure, treatment novelty, and clinical benefit—to explore conditions for prioritizing rare disease coverage. The second, unlabeled DCE, compared rare disease scenarios, adding QALY gains and social insurance premium increments to estimate willingness-to-pay per QALY and derive incremental cost-effectiveness ratio (ICER) thresholds. A D-efficiency design produced 16 and 20 choice sets, blocked into two versions. In 2023, 120 decision-makers were invited for online data collection via interviews. Analysis used conditional logit models to assess preferences.

**Results:**

In total, 101 eligible decision-makers provided complete responses and were included in the analysis. Nearly half of the respondents were female (47.5%), with 58.4 percent having over 10 years of professional experience. Many participants had expertise in health insurance system research (48.5%) and pharmacoeconomics (77.2%). The first DCE found rarity alone did not add value; decision-makers prioritized disease severity, drug innovation, and health benefits, especially the first two. The second DCE estimated an ICER threshold for rare diseases of 1.9 times the gross domestic product per capita—around three times China’s current baseline threshold—reflecting equity considerations.

**Conclusions:**

Chinese decision-makers demonstrated a willingness to assign higher value to rare disease treatments, particularly when considering severity and novelty. This study provided empirical evidence that supports higher ICER thresholds for rare disease treatments to account for equity considerations. The findings offer valuable insights for policymakers seeking to refine health insurance reimbursement strategies and ensure a more equitable allocation of resources.

